# A randomised controlled trial of a telephone administered brief HIV risk reduction intervention amongst men who have sex with men prescribed post-exposure prophylaxis for HIV after sexual exposure in the UK: Project PEPSE

**DOI:** 10.1371/journal.pone.0216855

**Published:** 2019-05-23

**Authors:** Carrie Diane Llewellyn, Charles Abraham, Alex Pollard, Christopher Iain Jones, Stephen Bremner, Alec Miners, Helen Smith

**Affiliations:** 1 Brighton and Sussex Medical School, Brighton, United Kingdom; 2 Faculty of Medicine, Dentistry and Health Sciences, University of Melbourne, Australia; 3 London School of Hygiene & Tropical Medicine, London, United Kingdom; 4 Lee Kong Chian School of Medicine, Nanyang Technological University, Singapore; Agencia de Salut Publica de Barcelona, SPAIN

## Abstract

**Background:**

In western countries, men who have sex with men (MSM) are most affected by HIV and increasingly likely to engage in risky sexual behaviour. MSM who experience a potential sexual exposure to HIV (PEPSE) and receive a preventative regimen of anti-HIV treatment are at particularly high risk of acquiring HIV and could potentially benefit from targeted risk reduction behavioural interventions such as motivational interviewing (MI).

**Purpose:**

The aim of this trial was to examine the impact of augmented MI (MI plus information provision and behavioural skills building), over and above routine care, on reducing risky sexual behaviour in MSM prescribed PEPSE. Secondary aims of the research were to examine whether the intervention reduced sexually transmitted infections (STI) and further requests for PEP.

**Methods:**

A parallel-group pragmatic randomised controlled trial was conducted with 175 MSM recruited from five sexual health (SH) clinics in the south east of England. The intervention was two fixed-duration sessions of telephone administered augmented MI. A manual guided the selection of individualised persuasive communication strategies based on underlying change mechanisms specified by the Information, Motivation and Behavioural Skills (IMB) model. Primary outcomes were the number of receptive and active anal intercourse (AI) partners, the use of condoms every time during receptive and active AI and the use of condoms sometimes during receptive and active AI.

**Results:**

There were no significant impacts on sexual risk behaviour or any of the psychological measures, and no discernible reduction in requests for repeat PEP or rates of STIs within a year.

**Conclusion:**

Our behavioural intervention of augmented MI did not affect risky sexual behaviour, rates of further PEP and STIs, and psychological factors, in MSM prescribed PEPSE.

**Trial registration numbers:**

UKCRN ID:11436; ISRCTN00746242.

## Introduction

Men who have sex with men (MSM) are the group most affected by the HIV epidemic in western countries [1,2] and their sexual risk taking behaviour is reported to be increasing [3,4]. Post-exposure prophylaxis (PEP) with anti-HIV treatment following potential sexual exposure (PEPSE) has been recommended as one method of preventing HIV infection in the UK [5]. As part of a comprehensive strategy for HIV prevention and care, behavioural interventions are an important tool in the global fight against HIV [6]. One-to-one behavioural interventions, such as motivational interviewing (MI) have been recommended [7,8] to reduce HIV in high risk groups. The UK’s National Institute for Health and Care Excellence (NICE) and Public Health England (PHE) guidance recommends one-to-one behaviour change interventions be provided within STI/HIV services [5,9], stating these interventions are integral to the modernisation of sexual health services [8]. Seeking PEP after potential sexual exposure may indicate an unmet prevention need and provides an opportunity to target interventions at a “teachable moment” to motivate individuals to explore recent risk behaviours thus potentially lowering the likelihood of further risk behaviour [10,11].

Behavioural risk reduction in clinical settings to prevent or reduce HIV are a public health priority, and clinic based interventions are under increasing pressure to be as brief as possible to optimise cost-effectiveness. The Information-Motivation-Behavioral Skills model (IMB) has been provided as a theoretical basis for a range of effective interventions to reduce sexual risk taking behaviour [12,13] and therefore, provides a useful logic model specifying modifiable behavioural antecedents [14].

The IMB model proposes that for individuals to manage their behaviour to prevent HIV prevention, they must have relevant information about how HIV is transmitted and methods of prevention (knowledge). Moreover, they must believe that they are at risk of HIV, have positive attitudes toward prevention, and positive social norms for prevention (that is, believing others approve of prevention). They must also have the necessary behavioural skills to enable them to discuss condom use and safer sex with partners, purchase and use condoms correctly. They must also be confident in their ability to use these skills effectively (high self-efficacy). The model further proposes that information relevant to the personal practise of preventive behaviour, motivation to practise prevention and behavioural skills for practising prevention effectively, are fundamental determinants of HIV/STI preventive behaviour [15,16].

Talk-based therapies, such as motivational interviewing (MI) [17,18], have routinely been used within sexual health (SH) settings to promote behaviour change [19]. MI is defined as a ‘directive, client-centred counselling style for eliciting behaviour change by helping clients to explore and resolve ambivalence’ [18]. It can be especially effective for individuals who are reluctant to change or who are ambivalent about changing their behaviour, and may be particularly appropriate for MSM who may benefit from more tailored preventive strategies. A systematic review and meta-analysis of RCTs showed that interventions based on the principles of MI outperformed traditional advice giving in the management of a range of behavioural problems and diseases [20]. More recently, a pilot study has shown the effectiveness of telephone-administered MI to reduce risky sexual behaviour of HIV infected people in rural populations in the US [21].

The aim of this study was to examine whether a brief behavioural intervention consisting of two-sessions of telephone administered augmented MI, (informed by the IMB), reduces risky sexual behaviour (compared with ‘treatment as usual’) in MSM prescribed PEP treatment after potential sexual exposure to HIV. The IMB approach has not previously been applied to a short term prophylactic regimen (PEP) of medication after risky sexual exposure but its successful application to HIV risk reduction suggests that it is an appropriate model on which to base an intervention for people who report sexual risks and are prescribed PEP. As MI-based interventions are commonly employed in SH settings, the brief intervention developed here has the potential to be acceptable, practical, sustainable and potentially cost-effective in this population [22]. A secondary aim of this research was to examine the impact on psychological determinants such as motivation to avoid risky sexual behaviours and risk reduction behavioural skills.

### Hypotheses

We hypothesized that compared with treatment as usual, those in the intervention arm would report a reduction in the proportion of sexual practices considered risky (less unprotected anal intercourse (UAI) (receptive and insertive), increased use of condoms, fewer partners); have lower incidences of STIs and HIV; have greater knowledge of risk reduction strategies; have greater motivation to avoid risky sexual behaviours and have improved risk reduction behavioural skills.   

## Materials and methods

### Study design

**Fig 1:**
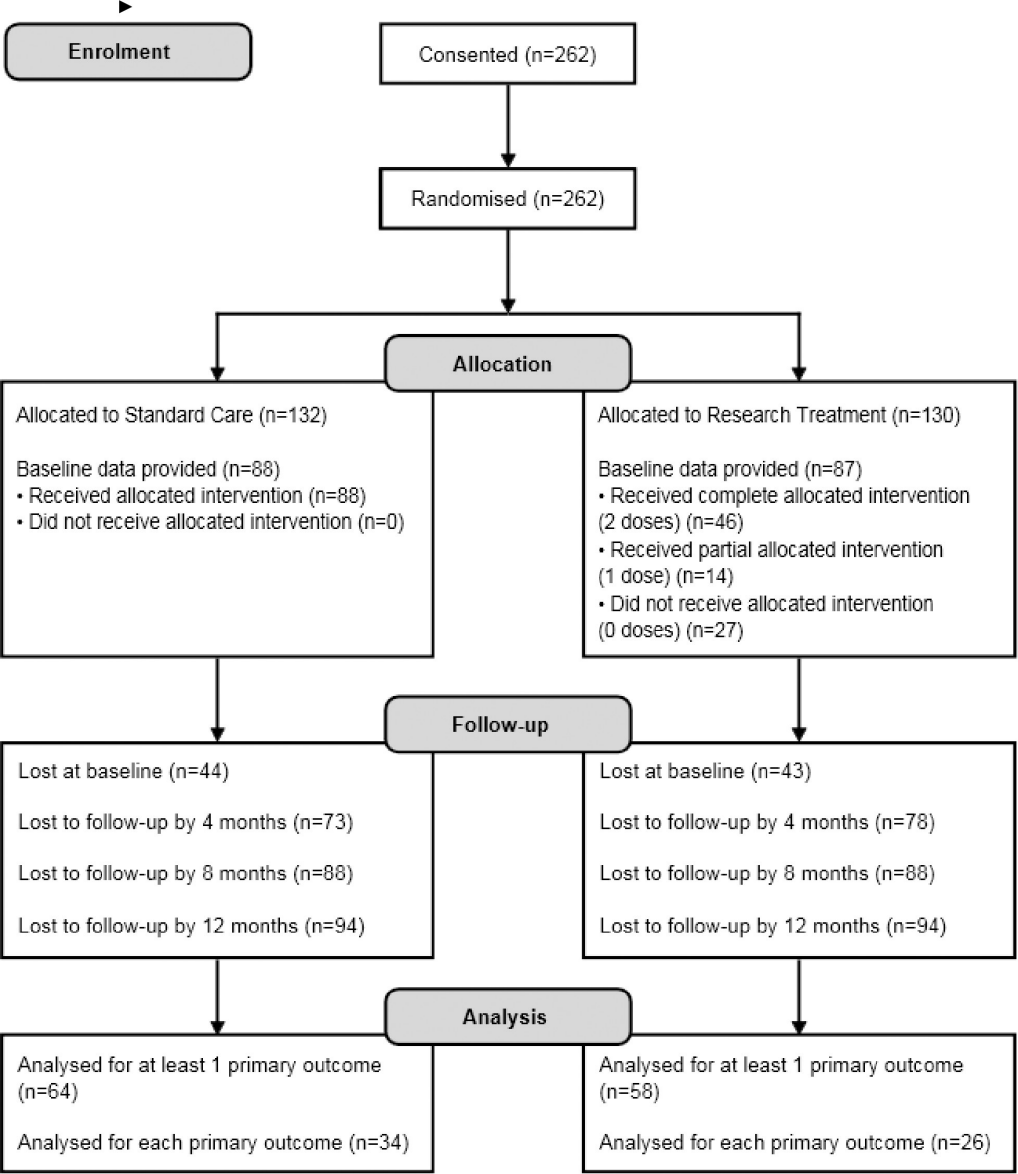
CONSORT flowchart of study participants.

Parallel group randomised controlled trial (Fig 1) [23]. Trial registration numbers: UKCRN ID:11436; ISRCTN00746242.

### Participants

#### Eligibility criteria

Participants were MSM, aged ≥16 years, attending a sexual health (SH) clinic for the prescription of PEP after sexual exposure, who were able to give written, informed consent.

#### Exclusion criteria

People with learning difficulties; those unable to read study materials; those with no means of communication; and those who were seeking PEP after sexual assault. In the original protocol [23] S1 and S2 Files, we had planned to exclude people who had previously received psychological support in relation to their sexual risk taking. However, we subsequently gained ethical amendment to remove this exclusion criterion as it proved unfeasible to implement and was not representative of everyday professional practice.

Ethical amendment was approved to alter the original analysis plan from mixed affects ANCOVAs to mixed effects negative binomial models and linear models to better take into account missing data and the original power calculation relates to the original protocol [23] S1 and S2 Files. We have not retrospectively applied a power calculation to the data [24].

### Recruitment procedure

The data collection plan was tailored to the standard patient care protocols stipulated by the British Association for Sexual Health and HIV (BASHH) guidelines for PEP [25], and the clinical processes in use at the recruitment sites. In the UK, patients are routinely prescribed an initial 5-day course of PEP medication in SH clinics, or at A&E departments out of hours. Whichever mode of access used, all patients then attend a dedicated PEP clinic at SH within 5 days of initial prescription for the remainder of their 28 day course of medication. Recruiting at PEP clinics ensured access to all patients irrespective of their mode of access. More detailed recruitment procedures can be found elsewhere [23].

### Randomisation and blinding

Participants were randomised on an individual one to one allocation ratio by an independent specialist company after consent. Randomisation occurred within each clinic using permuted blocks. This is a single-blind trial, as blinding of participants was not feasible. The statistician was blind to individual results during the trial and the blind broken at the time of analyses.

### Trial treatment arms

#### Control group: Routine care

Both the control and intervention arms received ‘routine care’. Patients were seen initially by a Health Advisor (HA) or Specialist Nurse (SpN) for consultation, a 5 day prescription of PEP and blood tests (HIV, Hepatitis B and liver function). Five days later patients received a first follow-up appointment for further PEP (if tests confirmed HIV–ve). On completion of the 28 day PEP treatment regimen patients received either a face-to-face or telephone appointment with a HA or SpN to discuss their sexual health, adherence to PEP and blood test results. At 4 months after exposure (3 months after the end of PEP) patients were routinely recalled to the clinic for HIV testing. Participants allocated to the control group were asked to complete all measures at the same time points as the intervention group.

#### Intervention group

In addition to ‘routine care’ the intervention group received two telephone sessions of augmented MI, with information and skills building based on the IMB model of behaviour change. The first telephone call was made after baseline assessments were received, the second call was made 7–14 days later. Telephone delivery enabled one person (AP) to conduct all intervention sessions, thus controlling for provider differences and facilitating recruitment from a wide geographical area in an economical manner.

### Duration and content of intervention

#### Telephone delivered MI

The telephone sessions were up to 30 minutes in length. The interventionist initially elicited individual risk behaviours and any informational, motivational or skill deficits which may have contributed to participants’ risky sexual behaviours and discussed particular areas related to risky sex e.g. the use of alcohol or drugs. The interventionist elicited self-motivational statements from participants through the use of open-ended questions and utilised MI-based strategies to increase motivation to change, including: 1) Providing the participant with feedback about his risky sexual behaviours; 2) Increasing the participant’s sense of responsibility to reduce risky sexual behaviours; 3) Providing brief and direct advice to create a desire for change; 4) Providing a menu of options from which the participant could choose to reduce risk; 5) Demonstrating empathy by listening carefully, and acknowledging an accurate understanding his problems; and 6) Enhancing self-efficacy to reduce risky sexual behaviours [31]. The second session contained similar content to the first but reiterated and built upon the risk reduction motivation from session 1.

A bespoke risk-reduction manual guided the selection of persuasive communication strategies appropriate for each participant and was based on underlying change mechanisms specified by the IMB theoretical framework (available on request from the authors). The manual described how to elicit information and included scripts describing likely sexual-risk scenarios relevant for MSM. Specific behaviour change techniques were identified [26,27] and possible responses exemplified.

#### Information provision

Information about HIV risk behaviour, prevalence and strategies to minimise risk were provided to those in the intervention arm in the form of informational booklets (*‘Ready for Action’* Second Edition and ‘*Get it on’* condom guide both produced for MSM by the Terrence Higgins Trust) (available on request from the authors). These were sent by email after baseline measures were returned and before the intervention. In session 1, the interventionist prompted the participant to read the information if they had not done so.

#### Skills building

Skills building component was delivered in the form of interventionist-provided suggestions of practical strategies needed to develop these skills. An individually-tailored action plan was developed and mutually agreed at the end of each session. The action plan had headings such as ‘*what you will do differently*, *at what point you will do it*, *and how much/how often’*, and contained ‘*I will* …’ statements. The sub-section ‘Making Plans’ included completing statements such as ‘*“My first step will be* …’. The final section asked for consideration of ‘*What might get in your way*, *and how do you plan to get past any problems or temptations…*?’ Examples included “***I will …*** *remember that men with HIV have good reasons why they may not always disclose their HIV status—so asking people their HIV status isn’t an effective way to avoid HIV**”**. **“I will …** reduce the chance of condoms breaking by*:*—finding condoms that fit (**www*.*freedoms-shop*.*nhs*.*uk**); checking on the condom and adding lube during long or particularly vigorous fucks”*. ***“I will …*** *by the end of this week*, *add text to all my cruising profiles stating that I am looking for safe and drug free sex only*, *and change the safe-sex statement to ‘Safe sex only’”*. The interventionist completed the action plan pro forma with the mutually agreed plans and sending this to the participant by email after the session. On the reverse side of the Action Plan a Certificate of Commitment was included with the following affirmative statements which participants were encouraged to sign. *‘I am my own person and I want the best for me*. *I intend to stay HIV Negative*, *and to save myself*, *and the people who care about me*, *from the damage HIV/AIDS would do to my life*. *I have people who care about me and a future to look forward to*. *I am in control and I am going to get what I want out of life*. *I promise to myself that I will…*.(to be completed by participant).

### Treatment fidelity

Assessing the fidelity of the treatment is an important component of successful research dissemination. In order to monitor the fidelity and validity of intervention delivery, assessment of both the interventionist and the participant were conducted according to National Institutes of Health (NIH) Behaviour Change Consortium ‘best practice’ recommendations. The intervention was limited to two sessions. Adherence to the treatment manual was optimised by training and monitoring and providing feedback during the intervention (from CA and BH). A validated instrument, the Motivational Interviewing Treatment Integrity (MITI) 3.1.1[28], was used to provide structured feedback, to monitor and document adherence to MI principles during weekly supervision. The interventionist was also required to complete a process evaluation checklist (adapted from the NHS Health Trainer Handbook [29]) after each intervention session to remind him to include the appropriate skills and content for each intervention and minimise bias. A retrospective process evaluation of the audiotaped recordings was conducted by an independent researcher.

### Measures

#### Socio-demographic characteristics

Participants were asked their age, sexual orientation, employment status, ethnic background and their highest educational qualification. Participants were also asked whether they were in a sero-discordant relationship with a main sexual partner, whether they had received any psychological support for sexual health matters, whether they had received money or favours for sex in the last 4 months and whether they used alcohol or drugs before having sex.

#### Primary outcomes

A number of reported risk behaviour outcomes were used to gauge potential HIV transmission-risk sexual behaviour as no single index adequately reflects the complex nature of HIV transmission risk: the proportion of different partners where unprotected anal intercourse (UAI) (receptive and insertive) took place in the previous four months with individuals of unknown or HIV positive status, and the consistency of condom use. Six questions were asked: 1. How many different people have you fucked (you were ‘active’ in anal sex) during the last four months? 2. With how many of these partners were condoms used *every time*? 3. With how many of these partners were condoms used *only sometimes*? 4. How many different people have you been fucked by (you were ‘passive’ in anal sex) during the last four months? 5. With how many of these partners were condoms used *every time*? 6. With how many of these partners were condoms used *only sometimes*? Questions 2 and 5 were used to denote consistent condom use, and questions 3 and 6 to denote inconsistent condom use. To ascertain relative consistency of condom use with different partners, and therefore risk, in both the active (insertive) and passive (receptive) roles, responses to questions 2 and 3, and 5 and 6 were divided by the responses to questions 1 and 4 respectively, to give percentages of partners where condoms were used either ‘all the time’ or ‘only sometimes’. The style of language and wording employed in the materials for this study were adapted from the annual Gay Men’s Sex Survey (GMSS), the longest continuously running survey of gay men’s sexual behaviour in the world with proven effectiveness and acceptability to MSM. All study materials (measures, training manual, intervention, method for data sharing) were piloted on a sample of MSM and refined in response to their feedback, together with input from our study’s lay Advisory Board and Steering Group.

#### Secondary outcomes

The incidence of subsequent STIs and HIV were self-reported, with a particular focus on anal gonorrhoea and/or Chlamydia as it is recognised these are directly related to condom use.

### Psychological determinants

#### Information

To measure levels of HIV risk-reduction information/ knowledge the ‘Health and Relationships Survey’ [30] was adapted for use with MSM. The measure consisted of six statements scored on a 5 point Likert scale (1 = Strongly agree). Example items include: *‘as long as you or your partner don’t ejaculate (cum) while fucking there is no need to use condoms*’; *oil based lubricants (e*.*g*. *Vaseline) are safe for use with condoms*’; ‘*I would believe a partner if they told me their HIV status*’. The HIV information scale is scored by dichotomising each item into a value of 1 (correct) or 0 (incorrect) and then summing the item values. For analysis of true items, responses of 1 or 2 (*agreement*) were recoded as 1, and all other responses (including missing values) were recoded as 0. For false items, responses of 4 or 5 (*disagreement*) were recoded as 1 and all other responses coded as 0. Possible scores ranged from 0 to 6.

#### Motivation

Levels of motivation to avoid risky sexual behaviours and perform HIV preventive behaviours were evaluated through measures of attitudes, subjective norms and behavioural intentions in relation to HIV risk-reduction. Items included: ‘talking about safer sex with a partner’, ‘persuading a partner to practice safer sex’, ‘having own supply of condoms’, ‘having condoms available at all times’, ‘always using condoms during anal sex’, ‘making condoms sexually exciting’, and ‘saying no or engaging in alternative safer sex acts if no condom is available’. For example, in relation to having condoms available at all times, we asked respondents to rate their performance on three 5-point semantic differential scales (*wise-foolish*, *feels good-feels bad*, *and overall good-overall bad*) adapted from Misovich’s Attitudes Towards AIDS Preventive Acts scale [30]. Ratings were reversed so that each item was scored from 1 (negative evaluation) to 5 (positive evaluation) and summed to provide a score of attitudes towards HIV preventive behaviours. Possible scores ranged from 15 to 75 with higher scores reflecting more positive attitudes towards safer sex behaviours. The subjective norm item for carrying condoms was; ‘*most people who are important to me think I should always have condoms with me*’. We also included measures of descriptive norms (e.g. *most of my gay friends will have condoms with them at all times’*), intentions (*‘I intend to always have condoms with me*’) and behavioural likelihood (likelihood to succeed) (e.g. *how likely is it that you will always have condoms with you over the next four months*?’). Behavioural likelihood was assessed using a five point response scale ‘likely to unlikely’, possible scores ranged from 5 to 25 with higher scores indicating more likelihood. All statements, except those assessing attitudinal constructs and behavioural likelihood, were assessed using five point Likert scales from ‘strongly agree’ to ‘strongly disagree’ and summed; possible scores ranged from 5 to 25 with higher scores indicating stronger endorsements.

#### Behavioural skills

Items in relation to the construct of action specific self-efficacy included: talking about safer sex with a partner, persuading a partner to practise safer sex, having own supply of condoms, having condoms available at all times, always using condoms during anal sex, making condoms sexually exciting, and saying no or engaging in alternative safer sex acts if no condom is available. For example, in relation to having condoms available at all times, we used the statement *‘I feel confident that I can have condoms with me at all times’*. Responses were made on five point Likert scales from ‘strongly agree’ to ‘strongly disagree’ and summed, possible scores ranged from 5 to 25 with higher scores reflecting higher self-efficacy and thus stronger behavioural skills. We included two items on Non-Specific Action Planning: *‘If I think having sex is likely*, *I plan ahead and think about what I need to do for safer sex’*, and *‘I have well thought out plans to make sure I only have safer sex’*. Responses were made on five point Likert scales from ‘strongly agree’ to ‘strongly disagree’ and summed, possible scores ranged from 2 to 10 with higher scores reflecting stronger non-specific action planning skills related to safer sex. Internal consistency for all of these measures was satisfactory.

Where relevant, measures used a retrospective recall period of ‘*the past 4 months’*. Other factors assessed by self-report questionnaire at each time point were alcohol and substance use. Socio-demographic (age, ethnicity, education, employment, relationship status) were assessed at baseline. The study protocol was approved by the National Research Ethics Service (NRES) Committee South East Coast—Surrey (ref: 11/LO/0718).

### Frequency and duration of follow-up

Primary and secondary outcomes were collected at baseline, 4 months (3 months after the end of treatment and intervention), 8 and 12 months. Follow-up questionnaires were accessed via an email link or the internet (as per consent) through a reminder (email or text).

### Analyses

The trial was planned, conducted and analysed on an intention to treat basis (i.e. according to the condition they were originally assigned to). No participants in the control group received the intervention.

Mixed effects negative binomial models were fitted for primary outcomes (Number of receptive /insertive anal intercourse partners in the last 4 months). Independent variables were intervention, number of partners (receptive/insertive) at baseline and time point. A random effect for patient was included to account for repeated measurements. Mixed effects linear models were fitted for primary outcomes (Use of condoms every time or some of the time in the last 4 months during insertive (active) anal intercourse); (Use of condoms every time or some of the time in the last 4 months during receptive (passive) anal intercourse) which were presented as proportions. Independent variables were intervention, proportion for each outcome at baseline and time point. p = 0.0083 was taken as the threshold for significance to account for the multiple co-primary outcomes.

To assess secondary outcomes, two logistic regression models were constructed, one was fitted with further PEP as the outcome and randomisation and number of partners at baseline as independent variables, and another logistic regression model was fitted with HIV/ Anal Gonorrhoea or Chlamydia diagnosis as the outcome and randomisation and number of partners at baseline as independent variables. To assess the effect of the intervention on psychological determinants, mixed effects linear models were fitted for the variables: Information, Attitude, Subjective norms, Descriptive norms, Intentions, Behavioural likelihood, Action specific self-efficacy and Non-specific action planning. Independent variables were intervention, baseline value of each outcome, and time point. Using complier average causal effect (CACE) principles [31], participants were defined as having received the intervention if they had received at least one dose. A random effect for patient was included to account for repeated measurements. All analyses were conducted using Stata 14.2 [32].

## Results

Recruitment took place between May 2011 and December 2012 from six clinical sites in the south east of England. Two-hundred and sixty-two men consented and of these, 175 (67%) provided baseline data. The mean age of the sample was 34.5 (SD = 9.1) years, (range 19 to 65). One hundred and sixty two (93%) participants were gay, 11 (6%) were bisexual, and <1% reported being heterosexual or would rather not say. Less than half the sample (46%) were of white British origin (n = 80), with a further 61 participants reporting as non-UK white (35%), 11 (6%) Asian, 7 (4%) Black African/Caribbean and 16 (9%) mixed race or ‘other’. The majority were in employment (81%) and 11% were students or unemployed/retired (8%). The majority (73%) were educated to at least degree level. Nineteen participants (11%) were in sero-discordant relationships with a main partner and 2 (1%) did not know the status of their main partner. Thirty-four (19%) reported having previously attended psychological support services in relation to their sexual health. Two extreme outliers were removed from baseline analysis, one man reporting 140 insertive partners and another reporting 75 receptive partners in the previous 4 months. The study team believed these were too extreme to be reliable.

The two groups were similar in terms of socio-demographic characteristics, previous use of psychological support for sexual risk taking, sero-discordancy with a main partner or receiving money or favours for sex (Table 1).

**Table 1:** Demographic and other relevant characteristics of participants at baseline (n = 175).

Characteristic	Intervention (n = 87)	Control (n = 88)
	n (%)	n (%)
Age in years (median, IQR)	34 (27–41)	32.5 (28–39)
Ethnicity:		
White UK	43 (49%)	37 (42%)
other	44 (51%)	51 (58%)
Employment:		
Self/employed	68 (78%)	73 (83%)
Student	11 (13%)	9 (10%)
Unemployed	6 (7%)	6 (7%)
Retired/other	2 (2%)	0 (0%)
Education:		
Below degree level	27 (31%)	21 (24%)
Degree	28 (32%)	42 (48%)
Post-graduate	32 (37%)	25 (28%)
Previous use of psychological support services	18 (21%)	16 (18%)
Sero-discordancy with main partner	10 (11%)	9 (10%)
Received money or favours for sex in the last 4 months	1 (1%)	3 (3%)

### Intervention compliance and attrition

Sixty (69%) participants in the intervention arm underwent telephone intervention with 46 (53% of the intervention arm) completing both doses. Similar rates of attrition were found across both arms (see CONSORT diagram Fig 1 and S3 File).

### Effect of intervention on self-reported behavioural risks

#### Number of partners

See Table 2 for descriptives of changes in outcome measures by treatment condition. No differences were detected between the intervention and control groups for the number of insertive (active) AI partners (incidence rate ratio (IRR): 1.24, 95%CI: 0.79 to 1.94) and the number of receptive (passive) AI partners (IRR: 0.74, 95%CI: 0.48 to 1.12) in the last 4 months at any time point.

**Table 2:** Changes in outcome measures by treatment condition.

OUTCOMES	CONTROL			INTERVENTION			OVERALL			IRR or Randomisation effect estimate coefficient	95% CI	P value
	Median	IQR	N	Median	IQR	N	Median	IQR	N			
**Number of insertive (active) AI partners**										1.24	0.79–1.94	0.36
**Baseline**	3.0	1.0–6.0	87	3.0	1.0–5.0	81	3.0	1.0–5.0	168			
**4 months**	1.5	0.0–3.0	58	2.0	0.0–5.0	51	2.0	0.0–5.0	109			
**8 months**	1.5	0.0–3.0	44	2.0	0.0–5.0	42	2.0	0.0–5.0	86			
**12 months**	1.0	0.0–3.0	38	1.0	0.0–3.0	36	1.0	0.0–3.0	74			
**Number of receptive (passive) AI partners**										0.74	0.48–1.12	0.15
**Baseline**	3.0	1.0–5.0	86	3.0	1.0–6.0	84	3.0	1.0–5.0	170			
**4 months**	2.0	1.0–4.0	59	2.0	1.0–3.5	52	2.0	0.0–4.0	111			
**8 months**	2.0	0.0–4.0	43	1.0	0.0–5.0	41	1.5	0.0–4.0	84			
**12 months**	1.0	1.0–4.0	37	2.0	0.0–4.0	36	1.0	1.0–4.0	73			
**Use of condoms every time during insertive (active) anal intercourse**										0.05	-0.10–0.19	0.52
**Baseline**	0.8	0.5–1.0	69	0.8	0.5–1.0	67	0.8	0.5–1.0	136			
**4 months**	1.0	0.5–1.0	42	1.0	0.4–1.0	36	1.0	0.5–1.0	78			
**8 months**	0.8	0.5–1.0	29	1.0	0.5–1.0	31	0.9	0.5–1.0	60			
**12 months**	0.1	0.0–1.0	23	0.8	0.5–1.0	24	0.7	0.0–1.0	47			
**Use of condoms sometimes during insertive (active) anal intercourse**										0.00	-0.11–0.11	0.93
**Baseline**	0.2	0.0–0.5	65	0.1	0.0–0.4	63	0.1	0.0–0.5	128			
**4 months**	0.0	0.0–0.3	42	0.0	0.0–0.2	36	0.0	0.0–0.2	78			
**8 months**	0.1	0.0–0.5	30	0.0	0.0–0.2	31	0.0	0.0–0.4	61			
**12 months**	0.0	0.0–0.2	24	0.1	0.0–0.5	24	0.0	0.0–0.5	48			
**Use of condoms every time during receptive (passive) anal intercourse**										-0.12	-0.24–0.17	0.09
**Baseline**	0.7	0.4–1.0	73	0.8	0.5–1.0	71	0.7	0.5–1.0	144			
**4 months**	1.0	0.5–1.0	47	1.0	0.5–1.0	36	1.0	0.5–1.0	83			
**8 months**	1.0	0.5–1.0	30	1.0	0.3–1.0	28	1.0	0.5–1.0	58			
**12 months**	1.0	0.0–1.0	29	0.8	0.5–1.0	25	0.8	0.1–1.0	54			
**Use of condoms sometimes during receptive (passive) anal intercourse**										0.05	-0.06–0.16	0.35
**Baseline**	0.2	0.0–0.5	72	0.1	0.0–0.5	68	0.1	0.0–0.5	140			
**4 months**	0.0	0.0–0.2	45	0.1	0.0–0.5	35	0.0	0.0–0.3	80			
**8 months**	0.0	0.0–0.5	30	0.0	0.0–0.2	27	0.0	0.0–0.3	57			
**12 months**	0.0	0.0–0.0	29	0.0	0.0–0.3	25	0.0	0.0–0.3	54			
**Secondary Outcomes**												
**Further PEP Within 12 months**										0.60	0.21–1.68	0.33
	**Yes**			**Yes**			**Yes**					
	12			9			21					
**STI/HIV Within 12 months**										0.99	0.33–2.93	0.99
	**Yes**			**Yes**			**Yes**					
	15			10			25					

#### Condom use

No differences were detected between the intervention and control groups for the use of condoms every time during insertive (active) AI (coefficient: 0.05, 95 CI: -0.10 to 0.19) and in the use of condoms sometimes during insertive (active) AI (coefficient: 0.00, 95%CI: -0.11 to 0.11) in the last 4 months at any time point. No differences were detected between the intervention and control groups for the use of condoms every time during receptive (passive) AI (coefficient: -0.12, 95%CI: -0.24 to .17) and the use of condoms sometimes during receptive (passive) AI (coefficient: 0.05, 95%CI: -0.06 to 0.16) in the last 4 months at any time point. For further analysis using complier average causal effect (CACE) principles, participants were defined as having received the intervention if they had received at least 1 dose. Results from the CACE analysis corresponded very closely with the results from the ITT analysis, with no difference between the intervention and control groups for any outcome.

### Secondary outcomes

#### Effect of intervention on further requests for PEP

Twenty-one participants were prescribed a further course of PEP within the 12 months of the study (n = 12 prescribed further PEP in the control group and n = 9 in the intervention group). There was no evidence of a difference in requests for further PEP between the intervention and control groups (OR = 0.60, 95%CI: 0.21 to 1.68).

#### Effect of intervention on self-reported STIs/ HIV

Within 12 months, 24 participants reported a STI: 14 in the control arm and 10 in the intervention arm. Three people were diagnosed with HIV within one year: two in the control arm and one in the intervention arm (Table 2). Although fewer STIs and HIV diagnoses were reported in the intervention arm, there were no statistically significant differences between arms (OR = 0.99, 95%CI: 0.33 to 2.93).

#### Effect of intervention on psychological determinants

See Table 3 for descriptive statistics on changes in psychological determinants by treatment condition. No difference was detected between the intervention and control groups on levels of Information (coefficient: 0.14, 95%Cl:-0.15 to 0.43); Attitudes (coefficient: -0.86, 95% CI: -3.25 to 1.54); Subjective norms (coefficient: 0.20, 95%CI: -0.74 to 1.14); Descriptive norms (coefficient: -0.73, 95%CI: -1.70 to 0.24); Intentions (coefficient: 0.21, 95%CI: -0.73 to 1.16); Behavioural likelihood (coefficient: -0.23, 95%CI: 1.19 to 0.74); self-efficacy (coefficient: -0.05, 95%CI: -0.85 to 0.75); or action planning (coefficient: 0.06, 95%CI: -0.41 to 0.53).

**Table 3:** Changes in psychological determinants by treatment condition.

Determinants (Cronbach’s α)	CONTROL			INTERVENTION			OVERALL			Randomisation effect estimate coefficient	95% CI	P value
	Median	IQR	N	Median	IQR	N	Median	IQR	N			
**I: Information** (α = .50)										0.14	-0.15–0.43	0.35
**Baseline**	4.0	3.0–5.0	84	5.0	4.0–5.0	84	4.5	3.5–5.0	168			
**4 months**	5.0	4.0–5.0	59	5.0	4.0–5.0	50	5.0	4.0–5.0	109			
**8 months**	4.0	4.0–5.0	41	5.0	4.0–6.0	41	5.0	4.0–5.0	82			
**12 months**	5.0	4.0–5.0	37	4.5	3.0–5.0	36	5.0	4.0–5.0	73			
**M: Attitude** (α = .89)										-0.86	-3.25–1.54	0.48
**Baseline**	69.0	64.0–75.0	87	71.5	67.0–75.0	84	71.0	66.0–75.0	171			
**4 months**	72.0	63.0–75.0	59	71.0	65.0–75.0	50	71.0	64.0–75.0	109			
**8 months**	71.0	66.0–75.0	43	73.0	67.0–75.0	42	72.0	67.0–75.0	85			
**12 months**	72.0	62.0–75.0	37	71.5	66.5–75.0	36	72.0	64.0–75.0	73			
**Subjective norms** (α = .80)										0.20	-0.74–1.14	0.68
**Baseline**	23.0	20.0–25.0	85	24.0	22.0–25.0	84	24.0	21.0–25.0	169			
**4 months**	23.0	21.0–25.0	59	25.0	22.0–25.0	49	24.0	21.0–25.0	108			
**8 months**	25.0	21.0–25.0	43	25.0	21.0–25.0	42	25.0	21.0–25.0	85			
**12 months**	25.0	21.0–25.0	39	24.0	20.0–25.0	36	24.0	20.0–25.0	75			
**Descriptive norms** (α = .81)										-0.73	-1.70–0.24	0.14
**Baseline**	19.0	16.0–24.0	87	20.0	16.0–23.0	83	19.5	16.0–23.0	170			
**4 months**	20.0	17.0–24.0	58	19.0	16.0–24.0	49	20.0	16.0–24.0	107			
**8 months**	20.0	17.0–23.0	42	19.0	16.0–24.0	42	20.0	16.0–24.0	84			
**12 months**	20.0	17.0–22.0	37	18.5	16.0–23.0	36	20.0	16.0–22.0	73			
**Intentions** (α = .77)										0.21	-0.73–1.16	0.66
**Baseline**	24.0	21.0–25.0	87	24.0	21.5–25.0	84	24.0	21.0–25.0	171			
**4 months**	24.0	20.0–25.0	59	24.0	23.0–25.0	49	24.0	20.0–25.0	108			
**8 months**	23.0	19.0–25.0	43	24.5	21.0–25.0	42	24.0	21.0–25.0	85			
**12 months**	24.0	21.0–25.0	38	24.0	19.5–25.0	36	24.0	20.0–25.0	74			
**Behavioural likelihood** (α = .71)										-0.23	-1.19–0.74	0.65
**Baseline**	23.0	20.0–25.0	87	23.0	21.0–25.0	84	23.0	21.0–25.0	171			
**4 months**	23.0	21.0–25.0	59	24.0	22.0–25.0	49	24.0	21.0–25.0	108			
**8 months**	23.0	20.0–25.0	43	24.0	21.0–25.0	42	23.0	20.0–25.0	85			
**12 months**	24.0	21.0–25.0	38	24.0	20.0–25.0	35	24.0	21.0–25.0	73			
**B: Action specific self-efficacy** (α = .76)										-0.05	-0.85–0.75	0.90
**Baseline**	23.0	20.0–25.0	87	23.0	20.0–25.0	84	23.0	20.0–25.0	171			
**4 months**	23.0	21.0–25.0	59	25.0	22.0–25.0	49	24.0	21.0–25.0	108			
**8 months**	24.0	20.0–25.0	43	24.0	21.0–25.0	42	24.0	21.0–25.0	85			
**12 months**	24.0	22.0–25.0	38	24.0	20.5–25.0	36	24.0	21.0–25.0	74			
**Non-specific action planning** (α = .95)										0.06	-0.41–0.53	0.81
**Baseline**	8.0	6.5–10.0	84	8.0	6.0–9.5	84	8.0	6.0–10.0	168			
**4 months**	8.0	7.0–10.0	59	9.0	7.0–10.0	50	8.0	7.0–10.0	109			
**8 months**	9.0	7.0–10.0	41	9.0	8.0–10.0	41	9.0	7.0–10.0	82			
**12 months**	8.0	8.0–10.0	37	8.0	8.0–10.0	36	8.0	8.0–10.0	73			

### Treatment fidelity

A random sample of 54 recordings out of 88 (61%) were transcribed using smart verbatim. These transcripts were then coded using the MITI 3.1.1 [28] to assess the interventionist’s ability to deliver MI effectively to participants. Transcripts were given a global score out of 5 for *Evocation*, *Collaboration*, *Autonomy/Support*, *Direction* and *Empathy*. In addition, the interventionist was assessed for behaviour counts. These behaviours include; *MI adherent behaviours*, *MI non-adherent behaviours*, *open* and *closed questions* and *simple or complex reflections*. Summary scores were then calculated. The analysis of the 54 transcripts show the mean scores (SD) for empathy 4.4 (0.61), global spirit rating 4.0 (0.48), percentage complex reflections 52.4% (13.10), percentage open questions 31.4% (11.26) reflection to question ratio 1.2 (0.54) and percentage MI adherent 92.7% (12.47). The interventionist achieved satisfactory proficiency in all scores apart from percentage open question score [28]. There was no statistically significant difference (after correction for multiple testing) between the two sessions received by participants apart from the score for Percentage Open questions; session 1 mean = 36.8% (16.46) compared to session 2 = 27.3% (13.46) (*p = 0*.*025)*.

## Discussion

The purpose of this study was to evaluate the impact of a brief intervention applying motivational interviewing techniques augmented with information and behavioural skills building (informed by the IMB Model), over and above usual care, on risky sexual behaviour in MSM prescribed PEP after potential sexual exposure. No difference between control and intervention groups was observed in relation to reported risky sexual behaviour, rates of further PEP prescriptions and STIs, and psychological antecedents. Thus we conclude that this brief, theory-based intervention did not enhance usual care.

### Limitations and strengths

Behavioural interventions aimed at reducing risky sexual behaviours related to HIV acquisition have been found to be moderately effective through systematic review and meta-analysis [33,34] and are frequently developed to target particular ‘risk’ groups. They vary considerably in content, theoretical underpinnings, delivery and target population [15,16,35]. Nonetheless, effective interventions often employ techniques to enhance levels of information, motivation, normative arguments, behavioural and interpersonal skills: specifically condom and safer sex negotiation skills as reported in the current study. We believe the use of a tried and tested underlying theory (MI and IMB) to guide intervention design is an important strength [36].

The British HIV Association PEP guidelines report that people may continue to put themselves at risk of HIV following PEP and repeatedly present for PEP [25]. A subsequent audit of PEP prescriptions amongst MSM during the period of this study (2009–2014) demonstrated that of 929 MSM receiving PEP at one clinic, 107 (11.5%) received more than one prescription within the five year period [37]. Repeat rates ranged from 2–9 with 321 prescriptions given within 5 years to this subgroup of 107 MSM. Seven patients seroconverted within the time frame of the audit. It is likely that within our current study group of patients prescribed PEP, we have a subsample of people who continue to experience high risks and may have experienced PEPSE prescription as a ‘wakeup call’ [38] or who were already practising safer sex behaviours but reported exceptional circumstances (excluding assault) [39]. For these men, seeking PEP is a rational strategy to mitigate exposure to HIV and to review any difficulties carrying out safer sex strategies. Although pre exposure prophylaxis (PrEP) was not recommended or available at the time of the study, many of these men experiencing repeat risks would now be candidates for discussions about PrEP [40], although recent research from Scotland where PrEP has been accessible since 2017 has also demonstrated an increase demand in PEPSE [41]. This clearly highlights the importance of other HIV risk reduction methods in combination with PrEP.

Scores on our measures were positively skewed so our assumption that this population would be of particularly high risk was incorrect. This is good news, from the perspective of PEP service delivery. It seems likely that in general PEP services do not need further behavioural intervention and are unlikely to benefit from brief interventions of the kind tested here.

It is noteworthy that 12% of our samples reported further PEP prescriptions during the 12 months of the study (which is a conservative estimate due to high drop-out) and there would be merit in identifying and targeting this particular multiple-PEP subgroup who continue to experience HIV risks after receiving PEP.

Other limitations include the large attrition rates seen at follow-up, although this is not unusual in evaluations of intervention sustainability over long time periods [42]. Much of the literature in this area documents short term sustainability. Positive (although not significant) trends noted in the outcome and psychological variables tended to diminish by 8–12 months. Whether this is due to attrition effects or lack of sustainability of any intervention effect over time is unknown. It is also possible that mere-measurement effects [43] of using a detailed questionnaire about sexual risk and behaviour on both arms obscured any intervention effect. The choice to complete a lengthy behavioural questionnaire about a personal subject may have led people in both arms to think in-depth about their sexual behaviour and under-report sexual risk.

Interventions of this kind places responsibility for change on vulnerable individuals which can be problematic for those experiencing the long term impact of marginalisation and stigma [44,45]. Negotiating safe sex is complex and theories which focus on the ways in which individuals’ behaviour during a sexual encounter is embedded within wider social contexts and syndemicy with other conditions may also be of benefit. Other authors have reported the co-concurrency of smoking, weekly binge-drinking, as well as historic experiences such as early sexual debut, illegal drug use, and less time in education, with sexual risk behaviours [46].

Analyses of the intervention transcripts showed that amongst a subsample of our participants, ‘chemsex’ (that is, sex involving simultaneous use of drugs) is implicated in some of the ongoing risks and that the reasons for drug use during sex is complex and heterogeneous [47]. A few brief sessions of a talking therapy to mitigate the impact of syndemic psychosocial conditions on vulnerability to HIV infection may not be beneficial in these situations and we would recommend offering additional sessions or recommending PrEP. The effects of alcohol are not directly specified in models such as the IMB [48] however the application of an motivational interviewing style intervention is flexible enough to accommodate these issues.

One of the strengths of this study is that it is the first to target people from a high risk group (MSM) at a potentially teachable moment (shortly after potential sexual exposure to HIV). It is the first sexual risk reduction intervention in the UK to deliver a telephone administered intervention to a clinic population, which has the potential to be easily administered and likely to be relatively inexpensive. Another strength is that although the intervention was tailored to the individual, it was also manualised so that the content was standardised, based on the principles of MI with the underlying change mechanisms specified in advance. We monitored the fidelity and validity of the intervention which was an additional strength of the study. The recruitment of MSM participants has been reported as the most challenging aspect of similar behavioural intervention studies [6]. To overcome these barriers we employed strategies to ensure that recruitment and retention were maximised, including: the refinement of assessment materials with the MSM community; the use of a telephone-based intervention to overcome geographic boundaries, time constraints and fear of public exposure; the recruitment of individuals seeking PEP rather than the recruitment of high risk individuals from the community.

### Implications for clinical practice and research

Despite the advent of pre-exposure prophylaxis (PREP) for HIV, there is potential benefit in providing behavioural interventions within clinical settings to reduce the sexual risk behaviour that accompanies HIV and STI transmission. Any such intervention would need to be implemented with little or no additional resources being provided to already-stretched sexual health services. The treatment fidelity findings show that a satisfactory proficiency level for the interventionist was obtained for all but one summary score. The fidelity results indicate that the intervention was delivered appropriately and with integrity. This has implications for sexual health advisors who could be used to deliver MI with proficiency when provided with the training to do so.

The study provides evidence that interventions of this nature are acceptable and engaging to this population which are important findings, although engaging participants in long term follow-up for research purposes is challenging. Amongst those that took part, feedback was positive. Although we did not conduct a cost-effectiveness analysis on these null findings, brief behavioural interventions of this kind delivered by telephone are likely to be cost effective and easily implementable within existing clinic structures, when effective. Future work targeting high risk repeat PEP users who do not uptake PrEP with a longer series of brief sessions addressing drug and alcohol use could help us understand the impact of behavioural interventions on safer sex behaviour.

### Conclusion

In conclusion, we did not demonstrate any impact of our behavioural intervention on risky sexual behaviour, rates of further PEP and STIs, and psychological factors, in a sample of MSM prescribed PEPSE. However, these findings still represent an important addition to the literature on how brief, theory-based behavioural interventions could enhance sexual services for MSM.

## Supporting information

S1 File Protocol. (PDF)

S2 File Published protocol. (PDF)

S3 File Consort checklist. (PDF)
