# Correction: A randomised controlled trial of a telephone administered brief HIV risk reduction intervention amongst men who have sex with men prescribed post-exposure prophylaxis for HIV after sexual exposure in the UK: Project PEPSE

**DOI:** 10.1371/journal.pone.0219998

**Published:** 2019-07-15

**Authors:** The PLOS ONE Staff

As a result of a technical error, the original article acceptance date is incorrect. The publisher apologizes for this error. The correct article acceptance date is May 1, 2019. The correct Received, Accepted, and Published dates for this article are as follows:

**Received:** January 5, 2018; **Accepted:** May 1, 2019; **Published:** May 23, 2019
