# Supplementary material for: A randomised controlled trial of a telephone administered brief HIV risk reduction intervention amongst men who have sex with men prescribed post-exposure prophylaxis for HIV after sexual exposure in the UK: Project PEPSE
Source: PLoS One. 2019 May 23;14(5):e0216855. doi: 10.1371/journal.pone.0216855 (PMC6532860; doi:10.1371/journal.pone.0216855)
Supplement: S1 File — (PDF) [file pone.0216855.s001.pdf]

Multicentre RCT and economic evaluation of a psychological intervention together with a leaflet to reduce risk behaviour amongst MSM prescribed post-exposure prophylaxis for HIV following sexual exposure (PEPSE)

PROJECT PEPSE

Co-applicants: Dr Carrie Llewellyn, Brighton & Sussex Medical School (BSMS)

Dr Martin Fisher, BSUH NHS Trust

Professor Charles Abraham, Peninsula College of Medicine & Dentistry

Professor Helen Smith, BSMS

Dr Paul Benn, Camden Provider Services at Central and North West London NHS Foundation Trust

Dr Matthew Hankins, BSMS

Dr Alec Miners, London School of Hygiene & Tropical Medicine(LSHTM)

Researcher/

interventionist: Mr Alex Pollard

Title: Multicentre RCT and economic evaluation of a psychological intervention together with a leaflet to reduce risk behaviour amongst MSM prescribed post-exposure prophylaxis for HIV following sexual exposure (PEPSE)

Project aims

Prevalence of HIV infections continue to rise in the UK, particularly amongst MSM and the reduction of new infections is a key component of the National Strategy for Sexual Health and HIV [1]. Recent reports on sexual health highlight the need to invest more in HIV prevention strategies [2, 3]. The Community HIV and AIDS prevention (CHAPS) policy (CHAPS is a partnership of several organisations led by the Terrence Higgins Trust) identifies post-exposure prophylaxis (PEP) as one HIV prevention strategy. Latest data shows that 59% of MSM in the South East Coast and 70% in London have heard of PEP, which is high compared to other regions [4]. Even higher levels of awareness are reported specifically in Brighton and Hove [5]. Local data from Brighton and the Mortimer Market clinics show an increase in the number people seeking PEP after sexual exposure.

The risk reduction intervention will consist of Motivational Interviewing (MI) augmented with information and behavioural skills building (informed by the Information-Motivation-Behavioral Skills Model). MI is defined as a 'directive, client-centred counselling style for eliciting behaviour change by helping clients to explore and resolve ambivalence' [6] which is particularly pertinent amongst MSM who may require a more tailored preventive strategy.

The Information, Motivation and Behavioural skills (IMB) model has been shown to provide a good basis for understanding and predicting HIV-relevant health behaviour and health behaviour change in almost two decades of research [7]. In addition, interventions based on this model have been effective in changing risky sexual behaviour among HIV negative and HIV infected individuals in more than 25 studies [8] and in changing levels of adherence behaviour amongst people with HIV/AIDS [9, 10]. The IMB has yet to be applied to a short term prophylactic regimen of medication after risky sexual exposure but its previous successful application to HIV risk reduction suggests that it is an appropriate model on which to base an intervention based on PEP. This will be the first time that a sexual risk reduction, telephone delivered intervention guided by the IMB model has been tested with individuals seeking PEP. This study will also provide estimates of the cost-effectiveness of the intervention through the inclusion of an economic evaluation.

Objectives and hypotheses

The primary aim is to examine the impact of motivational interviewing augmented with information and behavioural skills building (informed by the Information-Motivation-Behavioural Skills Model), over and above usual care, on risky sexual behaviour in MSM prescribed PEP after potential sexual exposure.

A secondary aim of this research is to examine the impact of the intervention on adherence to PEP.

Specifically we hypothesize that compared with treatment/ management as usual, those in the intervention arm will:

1) Report a reduction in the proportion of risky sexual practices (less unprotected anal intercourse (UAI) (receptive and insertive), increased use of condoms, a reduction in partners);

2) Have greater levels of adherence to PEP treatment;

3) Have lower rates of need (subsequent use) of PEP;

4) Have greater motivation to avoid risky sexual behaviours;

5) Have greater knowledge of risk reduction strategies;

6) Have greater risk reduction behavioural skills;

7) Have lower incidences of anal gonorrhoea;

8) Have lower incidences of HIV.

Background

Post-exposure prophylaxis following sexual exposure (PEPSE) to the human immunodeficiency virus (HIV) has been recommended as a method of preventing HIV infection in the UK [11]. Recommendations for treatment are derived from the existing use of antiretrovirals to prevent HIV infection after high risk occupational exposure to the virus ('needle-stick injuries') [12]. Recommendations for prescribing PEP result from the clinician's assessment of risk of transmission. If the risk of HIV transmission through particular sexual practices is of a similar magnitude as occupational exposures (such as unprotected anal intercourse and receptive oral sex) then PEP should be recommended [13].

Evidence suggests that PEP may reduce the risk of HIV infection if given within 72 hours and adhered to rigorously for 28 days [12, 14, 15]. Sustained adherence is required to prevent treatment failure, however, non-adherence of a quarter to a third of those prescribed it has been reported [5, 16]. The treatment can be challenging to patients due to side-effects such as diarrhoea, nausea, headaches and vomiting, and is costly.

Men who have sex with men (MSM) are the group most affected by the HIV epidemic in the UK [17] and their sexual risk taking behaviour is reported to be increasing [18, 19]. As part of a comprehensive strategy across HIV prevention and care, behavioural interventions remain an important tool in the global fight against HIV [20]. One to one behavioural interventions, such as motivational interviewing (MI) have been recommended [21, 22] to reduce HIV in high risk groups. NICE guidance places recommendations for one to one interventions within the context of current STI/HIV service provision [1, 23] and states that these interventions are integral to the modernisation of sexual health services [22].

Similar to repeat testers for sexually transmitted infections (STI's) and HIV, seeking PEP after potential sexual exposure may indicate an unmet prevention need and provides an opportunity to target interventions thus potentially lowering the likelihood of further risk behaviour [24, 25].

We will establish a telephone-administered intervention based on motivational interviewing (MI) augmented with information and behavioural skills building (informed by the Information-Motivation-Behavioral Skills Model). MI is defined as a 'directive, client-centred counselling style for eliciting behaviour change by helping clients to explore and resolve ambivalence.'[6]. It is viewed as particularly useful for individuals who are reluctant to change or who are ambivalent about changing their behaviour. A systematic review and meta-analysis of RCTs shows that MI outperforms traditional advice giving in the treatment of a range of behavioural problems and diseases [26]. More recently, a pilot study has shown the effectiveness of telephone administered MI to reduce risky sexual behaviour in HIV infected rural populations [27].

The study will examine whether a two-session, telephone administered augmented motivational interviewing intervention based on the IMB model reduces risky sexual behaviour (compared with 'treatment as usual'), in MSM prescribed PEP treatment after potential sexual exposure to HIV. Evidence suggests that the Information-Motivation-Behavioral Skills (IMB) approach could be used to explain sexual risk taking behaviour [28]. This model proposes that information relevant to the personal practice of preventive behaviour, motivation to practice prevention and behavioural skills for practicing prevention effectively, are fundamental determinants of HIV/STI preventive behaviour [29].

The Information, Motivation and Behavioural skills (IMB) model proposes an intervention development process involving (1) elicitation of client resources and needs and, (2) matching intervention content to existing need and behaviour change objectives,(3) implementation of information, motivation and skill development techniques interventions according to protocol and, finally, (4) evaluation. This approach has been found to be effective in changing risky sexual behaviour among HIV negative and infected individuals in more than 25 studies (see [8] for a review) and in changing levels of adherence behaviour amongst people with HIV [9, 10].

Methods

Study design

Parallel group randomised controlled trial.

Participants

Eligibility criteria: Participants will be MSM, aged ≥16 years, prescribed PEP after sexual exposure, and attending a Genito-urinary Medicine (GUM) clinic who are willing and able to give written, informed consent.

Exclusion criteria: The following groups of patients will be excluded: people who have received previous psychological support from a clinical psychologist in relation to their risk taking; people with learning difficulties; or unable to read study materials; or with no means of communication acceptable to the patient; or who are seeking PEP after sexual assault.

Recruitment procedure

The data collection plan is tailored to the standard patient care protocols stipulated in the BASHH guidelines for PEP, and the clinical processes in use at the recruitment sites. PEP is available from GUM or via Accident & Emergency (A&E). Patients are routinely prescribed a 5-day course of PEP medication at general GUM clinics, or at A&E departments outside of GUM hours. Patient access routes then merge at dedicated PEP clinics at GUM within 5 days of initial prescription. Recruiting at the GUM clinic will ensure all patients who are prescribed the full course of PEP (28 days) are included in the trial from either access route. Eligible participants will be identified by a research nurse/ health advisor at the GUM clinic and given study information and consent forms.

Participants will have a 2-week window in which to consent and return baseline measures. This two week period allows the recruitment of patients at an early stage in their prescription, but at a time when their immediate anxiety should be reduced and they are able to give considered, informed consent.

Baseline and follow-up assessments

Participants enrolled into the study will be asked to self-complete a questionnaire at four time points during the study (at 0,3,6,12 months). The baseline questionnaire will be administered at the recruitment stage, and upon receiving the consent forms and baseline assessments, participants will be randomly allocated to either intervention or control conditions (see randomisation section).

The second questionnaire will be posted to patients or accessed via email/the internet (as per consent) prior to their 3-month follow-up appointment with the clinic. Any patients who have not returned completed questionnaires before their '3-month' follow-up will be sent repeat copies[1] (as per consent), and subsequently called on the telephone (if consented), and finally, may be approached by study staff at their clinic appointment.

The third questionnaire will be posted to patients or accessed via email/the internet (as per consent) 6 months after the end of PEP. Patients not returning their questionnaires will be sent repeat copies, and subsequently called on the telephone (as per consent). Those with a '6-month' follow-up appointment (depending on the recruitment site) and who have failed to return their questionnaire will be approached by study staff at their clinic appointment.

[1] Maximum of 2 repeat copies and 1 text/call/email if consented by patient.

The fourth questionnaire will be posted to patients or accessed via email/the internet (as per consent) 12 months after the end of PEP. Repeat copies, and subsequent telephone calls, will be used as reminders.

The recruitment period is for 12 months to allow sufficient numbers of participants to be recruited. A sample size of 250 (allowing for a 75% recruitment rate and 50% retention rate) is achievable within the time-frame and we anticipate that numbers will be higher given the increasing trends for PEP requests.

Randomization and blinding

The random allocation sequence will be computer generated in advance of the trial. Participants will be randomised after consent and collection of baseline measures. The recruiting staff will not have access to the results of randomisation prior to recruiting the participant. AP will be given the results of the randomisation on a case by case basis by a custodian who is unconnected to the project. Randomisation will occur within each clinic. A statistician (MH) will generate the allocation sequence (who will be blind to the identity of the individuals involved). This is a single-blind trial, given the nature of the behavioural intervention, blinding of participants is not feasible and the interventionist (AP) will know that all those he contacts are in the intervention arm. The PI and statistician will be blind to individual results during the trial and the allocation-to-trial-arm coding will be revealed when the dataset is sealed.

The interventionist (AP) and AP's supervisors will be blind to the baseline and follow-up measures which will not inform the delivery of the intervention. Anonymised measures will be entered onto the database by a person unconnected to the project.

Trial treatment arms

Control group: Treatment/management as usual

Both of the groups will receive ‘treatment/management as usual’: Patients are initially seen by a HA/SpN for an initial consultation, 5 day prescription of PEP and blood tests (for HIV, Hepatitis B and liver function). Patients receive their first follow-up appointment 5 days later to receive further PEP (if HIV–ve). After the 28 day treatment regimen patients receive either a face-to-face or telephone appointment with the HA/SpN to discuss their sexual health, adherence to PEP and the blood test results. At 4 months after exposure (3 months after the end of PEP) patients are recalled by the HA for HIV testing. In order to assess the impact of the intervention on outcome measures and IMB constructs, those allocated to control group 1 will be asked to complete all measures at 0,3,6, and 12 months.

Intervention group

Procedure

The intervention group will receive ‘treatment and management as usual’ plus the addition of an intervention which will be delivered as two telephone sessions employing motivational interviewing (MI) augmented with information and skills building based on the IMB model of behaviour change. The first call will be made within one week of the participant being consented into the trial and after baseline assessments are received. The second call will occur 7 days (+/-2 days) later. The intervention will be completed by the end of the 28 day course of PEP. A telephone format is proposed to allow the same person to conduct all interventional sessions to control for provider differences and to facilitate recruitment from a wide geographical area in an economical manner. If shown to be effective, this intervention would be sustainable within an improved routine care model.

Duration and content of intervention

Telephone delivered MI

Each telephone session will be a maximum of 30 minutes long. The second session will contain similar content to the first but will reiterate and build on the risk reduction motivation from session 1. In the case of drop-out between the 2 intervention sessions, a dose-response can then be assessed.

The interventionist will initially assess individual risk behaviours and any informational, motivational or skill deficits which have contributed to maintenance of participants' risky sexual behaviours and discuss particular areas related to risky sex e.g. the use of alcohol or drugs during sex. The interventionist will elicit self-motivational statements from the participant with the use of open-ended questions and will utilise MI strategies to increase motivation to change, including:

1) Providing the participant with feedback about his risky sexual behaviours

2) Increasing the participant's sense of responsibility to reduce risky sexual behaviours

3) Providing brief and direct advice to create a desire for change

4) Providing a menu of options from which the participant can choose to reduce risk

5) Demonstrating empathy by listening carefully, and accurately understanding his problems

6) Enhancing self-efficacy to reduce risky sexual behaviours [31]

If an individual discontinues with PEP before the end of the treatment, they will still be eligible to continue in the study. Data on adherence will be captured by the measures and a review of the medical notes.

The role of the manual

The treatment manual will guide the selection of persuasive communication strategies as appropriate for each participant and will be based on underlying change mechanisms specified by the IMB theoretical framework. This has been developed with particular input from the research fellow (AP), current PEP health advisors, co-applicants CA, CL and collaborator BH (with input from all co-applicants). The manual addresses steps to eliciting information, scripts for likely scenarios and responses and techniques to bring the sessions to a close.

Information provision and skills building

Information about HIV risk behaviour, prevalence and strategies to minimise risk will be provided to those allocated into the intervention arm ('Ready for Action' Second Edition, and 'Get it on' condom guide both produced by the Terrence Higgins Trust). This will be either sent by post, email or accessed via the internet (as preferred) after baseline measures have been returned but before the intervention. The interventionist will prompt the participant to read the information if they have not already done so.

The skills building component of the intervention will be given during the telephone session in the form of interventionist provided information, and practical strategies needed to master these skills. An action plan will be developed and agreed on (between interventionist and participant) during the session and the participant will be asked to write this down and practice. The interventionist will also write down the agreed action plan and send this to the participant after the session (either by post,or email as indicated by participant). Questions about adherence to practicing the action plan will be included in the follow-up questionnaires.

Vulnerable patients and disclosures

If during the intervention the patient discloses current self-harming thoughts or behaviour towards themselves or another, they will be referred to their treating consultant, GP or psychiatric support provided by the GUM clinic (we will have a list of contacts for each referring centre). The breaking of confidentiality under these circumstances is detailed on the consent form.

Anonymity and confidentiality of data

Participants anonymity will be protected by the allocation of a code (not a clinic or NHS number) for use on the database. Codes will be kept in a locked cupboard in the Department of Primary Care and Public Health, BSMS. Confidentiality of the data will be ensured by adhering to the Data Protection Act.

Confidentiality and data sharing between participant sites

All patient related information to be shared through encrypted NHS email system.

Interventionist training and supervision

The intervention will be delivered by a research fellow with experience of working with MSM populations in relation to sexual health and with experience of delivering motivational interviewing and educated to masters level. We have also costed in a 6 day advanced course in using MI with respect to sexual health run by Zest Consultancy (Members of the International Motivational Interviewing Network of Trainers), to refresh skills. CL is trained in motivational interviewing and will provide day to day supervision to the researcher. Additional supervision will be provided on a consultancy basis by a Consultant Clinical Psychologist (BH) who has extensive expertise of MI. The intervention based on the IMB Model will be developed with input from the steering group but particularly with expertise from CA and BH and piloted.

Treatment fidelity

Assessing the fidelity of the treatment is an important component of successful research dissemination. Translating effective behavioural change interventions from this research setting to clinical practice can be facilitated better when treatment fidelity strategies are used as guidelines for implementing new interventions in the clinic.

In order to monitor the reliability and validity of the intervention, assessment of both the interventionist and the participant will conducted as per National Institutes of Health (NIH) Behaviour Change Consortium 'best practice' recommendations. Ensuring same treatment dose within conditions will be ensured by a fixed number of sessions and by delivery within the intervention period (see intervention section). Ensuring interventionist skill acquisition and minimising 'drift' in interventionist skills will be minimised by the development and use of a treatment manual; by monitoring and providing feedback to the interventionist; and by providing adequate training. The gold standard method to ensure standard delivery of the intervention is to evaluate or code the sessions (through audiotape) according to predefined criteria. A validated instrument, the Motivational Interviewing Skill Code (MISC) [30], will be used to provide structured feedback, to monitor and document adherence to MI principles during weekly supervision. The interventionist will also be required to complete a process evaluation checklist (adapted from the NHS Health Trainer Handbook [31]) after each intervention session to remind them to include the appropriate skills and content for each intervention and minimise bias. The advisory board will be used to determine whether the treatment protocol has been adhered to during the recruitment and intervention period. Reduction of differences within treatments will be ensured by the use of one interventionist.

Should the valuation of the treatment delivery be found by BH to reduce, the researcher would undergo refresher training and mentoring with BH/CA.

Measures

Primary outcome: The proportion of risky sexual practices.

Risk Behaviour outcome measure

A Risk Behaviour outcome measure has been developed to include items of potential HIV transmission-risk sexual behaviour: number of episodes of unprotected anal intercourse (UAI) (receptive and insertive) over a three month time period with individuals of unknown or HIV positive status, consistency of condom use, number of partners using no protection.

(Unprotected receptive oral sex with an HIV+ve person is associated with minimal HIV transmission risk and thus is not included).

Secondary outcomes (and method of assessment):

i) Levels of adherence to PEP treatment (Morisky Medication Adherence Scale (MMAS) [32] adapted for use in PEP)

ii) Number of subsequent courses of PEP (self-report and medical record review).

iii) Levels of motivation to avoid risky sexual behaviours ('Measures of Motivation to Perform AIDS Preventive Behavior' questionnaire [33] adapted for use with MSM. This questionnaire provides assessment of attitudes, subjective norms and behavioural intentions in relation to HIV risk-reduction).

iv) Levels of HIV risk-reduction information/ knowledge ('Health and Relationships Survey' [33] adapted for use with MSM. Items are summed to form an HIV prevention information scale score).

v) Levels of risk reduction behavioural skills ('Behavioral Skills Measure' [33]. This consists of two subscales which assess the perceived difficulty of reducing HIV risk behaviour and the perceived effectiveness of methods to reduce risk).

vi) Diagnosis of anal gonorrhoea[2]

vii) Diagnosis of HIV.

With the exception of adherence, all outcome measures will use a retrospective recall period of 'the past 3 months'.

Other factors assessed by self-report questionnaire at each time point will be alcohol and substance use. Socio-demographic (age, ethnicity, education, employment, relationship status) will be assessed at baseline. Experience of treatment side-effects will be assessed using a previously used measure at 3 month together with the measure of adherence.

Frequency and duration of follow-up

The primary and secondary outcomes ii, iii, iv, and v will be collected at 3, 6 and 12 months after the end of treatment (and the end of the intervention). Secondary outcome i will be collected at 3 months only.

The follow-up period is for 12 months to determine intervention sustainability. Participants will be contacted by telephone prior to each follow-up assessment to remind them of their participation in the study and to ask their preference for follow-up assessment (postal, email/internet or via clinic attendance).

[2] Incidence data on rectal gonorrhoea will be collected as this is directly related to the risky sexual behaviour that we are assessing in this trial i.e. directly related to condom use.

Barriers to implementation

A recent review [20] highlighted that the recruitment of MSM participants was the most challenging aspect of similar behavioural intervention studies. To overcome these barriers we are employing strategies to ensure that recruitment and retention are maximised, including: the refinement of assessment materials with the MSM community; the use of a telephone-based intervention to overcome geographic boundaries, time constraints and fear of public exposure as an MSM; the recruitment of individuals seeking PEP by the HIV nurse specialist rather than the recruitment of high risk individuals from the community.

Analyses plan and power calculation

For the analysis of main effects for the primary outcome a mixed-design ANCOVA will be used with one between-groups factor (treatment vs. control) and one within-groups factor (baseline, 3-,6- and 12-month measures). For this analysis the estimated effect size is f=0.2 (based on a moderate estimate of effect size from a meta-analysis [24] of one to one interventions to reduce UAI in MSM). 90% power at 0.05 level of significance requires a sample of 250 (125 in each arm). Secondary outcomes will be analysed for the same effect size but with significance adjusted to 0.01 and power to 80%.

Piloting

All study materials (measures, training manual, intervention, method for data sharing) will be piloted in Phase 1 (see Gantt chart) on a sample of MSM and refined in response to their feedback, together with input from the Advisory Board and Steering Group. Application for ethics amendment may be required at this stage.

Economic evaluation

The economic evaluation will compare NHS costs of the intervention with usual care. Health outcomes will be expressed as quality-adjusted life-years (QALYs). Decision modelling techniques will be used to extrapolate the RCT results to predict longer term costs and health outcomes. A single economic model with an appropriate time horizon is proposed based on Bernoulli techniques (see Shepherd et al for a methodological explanation). This approach has been used in a number of previously published studies (Shepherd et al). This is a conservative approach because it only partially accounts for chains of infections which may be prevented, rather than prevention of single infections.

The purpose of the Bernoulli model is to translate changes in sexual behaviour as recorded in the trial into the probability of HIV transmission in order to estimate the number of averted transmissions. To inform this model, baseline data will be collected to allow a more complex infectious diseases model to be built if the intervention is proven. The data are likely to be useful for a range of other future HIV modelling studies for which further funding will be secured. The intervention costs will be collected contemporaneously. The longer term costs and health effects of HIV infection will be estimated using published evidence since infection-associated sequelae will not occur during the trial period. Future costs and QALYs will be discounted at 3.5% per annum. Results will be reported as incremental cost per QALYs and cost-effectiveness acceptability curves.

Patient and public involvement (PPI)

As part of the development of this grant application, MSM who were previous and current users of PEPSE were consulted using interviews during two clinic visits in Brighton in order to ask their opinions concerning: the proposed research design; the likelihood that they would take part in the research given the design and likely uptake of the brief telephone intervention if they were to be offered it as usual care in the future. We have also conducted in-depth interviews with users of PEPSE as part of earlier pilot work [34] which has directly lead to the development of this proposal.

We have also built into the proposal at an early stage an advisory board to include two lay members from the MSM community and a representative from the Terrence Higgins Trust. We have experience of recruiting and running an advisory board with lay members in our current research on sexually transmitted infections and have found lay members valuable. The advisory board will have collaborative, ongoing input into the design and management of the research and undertaking the research. Group meetings will be held at 4 key stages of the research. Subsequently, lay members will have direct input into the research which will be particularly informative during the refinement of the questionnaires and the dissemination of the research findings. The dissemination of the research findings to the MSM community will be guided/led by our advisory board member from the Terrence Higgins Trust.

In addition, the results of the study will be communicated to the public. At a local level, the gay community will be invited to a presentation of the study findings. This has been used successfully after previous community research studies on STIs run by MF and has generated feedback to enhance successive projects.

Dissemination

Research participants

Patients recruited into the trial will be asked whether they want access to the study report at the end of the study and this will be sent to them in electronic or paper form as preferred.

Local community

The local gay community will be invited to a presentation of the study findings. This has been used successfully after previous community research studies on STIs run by MF and has generated feedback to enhance successive projects. This is in line with the principles of 'Public Engagement in Science'. A well utilised infrastructure for information sharing exists with Brighton's organisations working with gay men. This includes meetings, newsletters and an annual publication of research outcomes.

National organisations and wider healthcare community

The results of the study would be communicated to national organisations such as The British Association for Sexual Health and HIV (BASHH), Genitourinary Nurses Association, Sexual Health Advisors Society and also to the voluntary sector via the Community HIV and AIDS Prevention (CHAPS) Initiative, which has a website for the use of partners and affiliate organisations.

Academic community

The findings will also be disseminated to academic audiences through publications in peer-reviewed journals (e.g.Health Psychology, British Medical Journal, JAIDS) and presentations at national (British HIV Association (BHIVA) and British Psychology Society conferences) and international conferences (International AIDS Conference, European Health Psychology Society Conference). Findings will also be disseminated via local meetings to health care professionals (HIV clinicians, specialist nurses, health advisors and pharmacists etc) in the field. We have costed publishing in open access journals and presenting at conferences.

Figure 1: The Information-Motivation-Behavioral Skills Model (Fisher and Fisher, 1992)

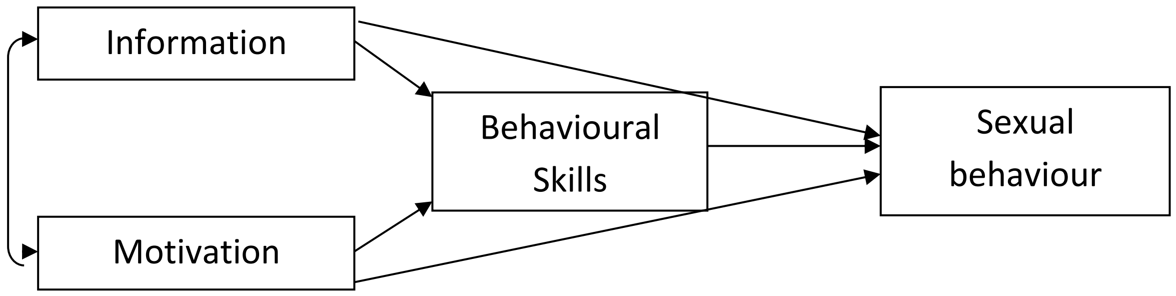

Figure 2: CONSORT flowchart of study participants

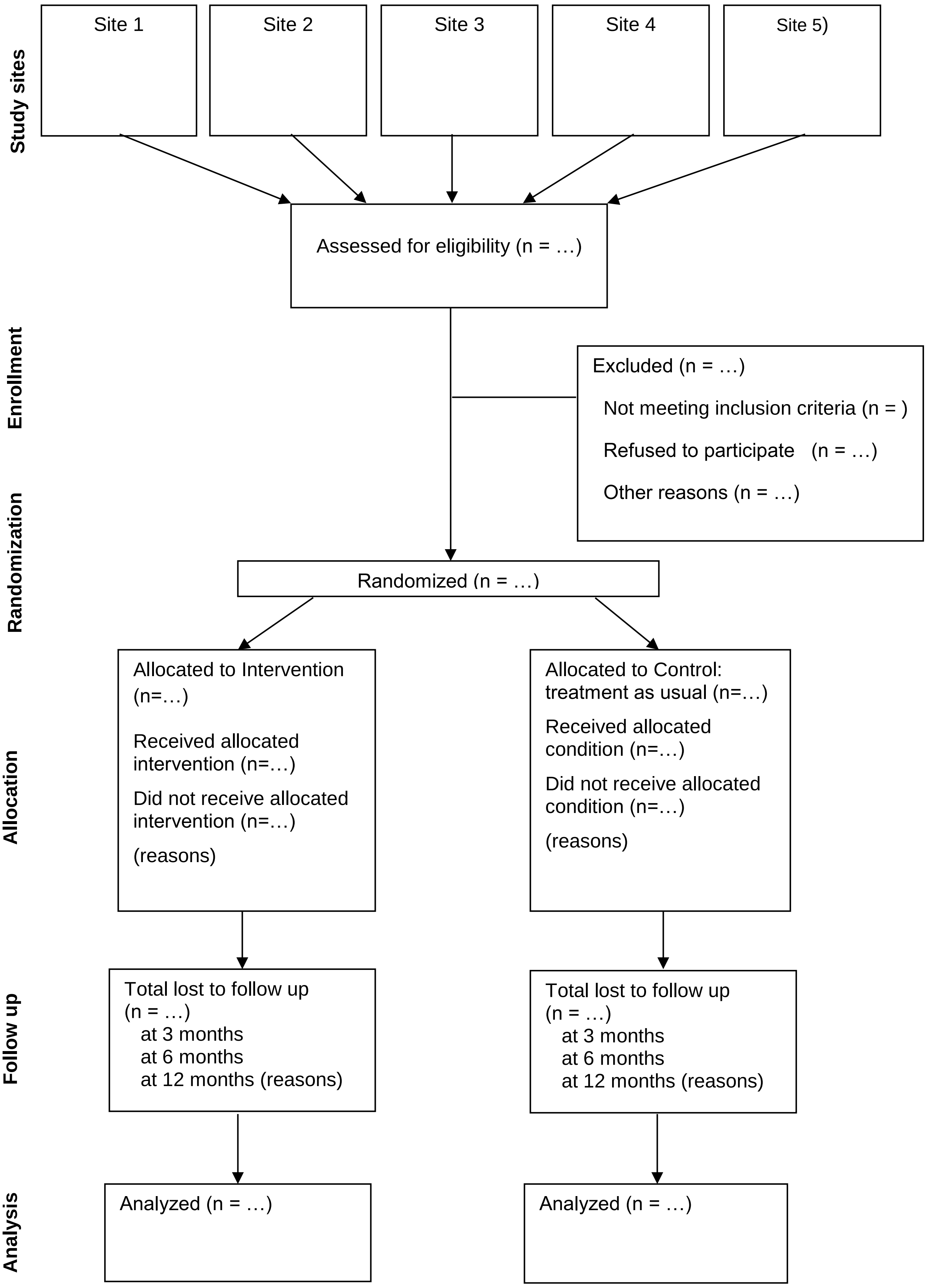

References

1. Department of Health. National Strategy for Sexual Health and HIV. London: DOH; 2001.

2. Department of Health. Moving forward: progress and priorities -working together for high quality sexual health: Government response to the Independent Advisory Group's review of the Sexual Health and HIV Strategy. London: Department of Health; 2009.

3. Independent Advisory Group on Sexual Health and HIV. Building on progress. Enhancing the response to HIV in England. London: Department of Health; 2009.

4. Hickson F, Weatherburn P, Reid D, Jessup K, Hammond G. Testing targets. Findings from the United Kingdom Gay Men's Sex Survey 2007. London: Sigma Research; 2009.

5. Dodds C, Hammond G, Keogh P, Hickson F, Weatherburn P. PEP talk. Awareness of, access to post-exposure prophylaxis among Gay & Bisexual men in the UK. London: Sigma Research; 2006.

6. Miller W, Rollnick S. Motivational interviewing, preparing people to change addictive behavior. New York: The Guildford Press; 1991.

7. Fisher JD, Fisher WA. The information-motivation-behavioral skills model. In: DiClemente R, Crosby R, Kegler M, editors. Emerging Theories in Health Promotion Practice and Research. San Fransisco: Jossy-Bass; 2002. p. 40-70.

8. Fisher JD, Cornman DH, Norton WE, Fisher WA. Involving behavioral scientists, health care providers, and HIV-infected patients as collaborators in theory-based HIV prevention

and antiretroviral adherence interventions. Journal of Acquired Immune Deficiency Syndromes 2006;43(Supplement 1):S10-S17.

9. Fisher JD, Fisher WA, Amico KR, et al. An information-motivation-behavioral skills model of adherence to antiretroviral therapy. Health Psychology 2006;25:462-73.

10. Starace F, Mayer KH, Amico KR, et al. Adherence to antiretroviral therapy: an empirical test of the information-motivation-behavioral skills model. Health Psychology 2006;25:153-62.

11. Fisher M, Benn P, Evans B, Pozniak A, Jones M, MacLean S, Davidson O, Summerside J, Hawkins D, Clinical Effectiveness Group (British Association for Sexual Health and HIV). United Kingdom Guidelines for the use of post-exposure prophylaxis for HIV following sexual exposure. International Journal of STD & AIDS 2006;17:81-92.

12. Cardo DM, Culver DH, Ciesielski CA, Srivastava PU, Marcus R, Abiteboul D, Heptonstall J, Ippolito G, Lot F, McKibben PS, Bell DM. A case-control study of HIV seroconversion in health care workers after percutaneous exposure to HIV-infected blood: clinical and public health implications. New England Journal of Medicine 1997;337(21):1485-90.

13. Katz M, Gerberding J. Postexposure treatment of people exposed to the HIV virus through sexual contact or injection-drug use. New England Journal of Medicine 1997;336:1097-100.

14. Otten RA, Smith DK, Adams DR, Pullium JK, Jackson E, Kim CN, Jaffe H, Janssen R, Butera S, Folks TM. Efficacy of postexposure prophylaxis after intravaginal exposure of pig-tailed

macaques to a human-derived retrovirus (human immunodeficiency virus type 2). Journal of Virology 2000;74(20):9771-5.

15. Schecter M, do Lago RF, Mendelsohn AB, Moreira RI, Moulton LH, for the Praça Onze Study Team. Behavioral impact, acceptability, and HIV incidence among homosexual men with access to postexposure chemoprophylaxis for HIV. Journal of Acquired Immune Deficiency Syndromes 2004;35(5):519-25.

16. Kahn JO, Martin JN, Rowland ME, Bamberger JD, Chesney M, Chambers D, et al. Feasibility of Postexposure Prophylaxis (PEP) against Human Immunodeficiency Virus Infection after sexual or injection drug use exposure: The San Francisco PEP Study. Journal of Infectious Diseases 2001;183(5):707-14.

17. The UK Collaborative Group for HIV and STI Surveillance. Mapping the issues. HIV and other sexually transmitted infections in the United Kingdom. London: Health Protection Agency Centre for Infections; 2005.

18. Dodds JP, Mercey DE, Perry JV, Johnson AM. Increasing risk behaviour and high levels of undiagnosed HIV infection in a community sample of homosexual men. Sexually Transmitted Infections 2004;80:451-4.

19. Elford J, Bolding G, Davis M, Sherr L, Hart G. Trends in sexual behaviour among London homosexual men 1998-2003: implications for HIV prevention and sexual health promotion. Sexually Transmitted Infections 2004;80:451-4.

20. Herbst JH, Beeker C, Mathew A, et al. The effectiveness of individual-, group-, and community-level HIV behavioral risk-reduction interventions for adult men who have sex with men: A Systematic Review. American Journal of Preventive Medicine 2007;32(4S):S38-S67.

21. Global HIV Prevention Working Group. Behavior Change and HIV Prevention: (Re) Considerations for the 21st Century. 2008. Global HIV Prevention Working Group.
Ref Type: Report

22. National Institute for Health and Clinical Excellence. One to one interventions to reduce the transmission of sexually transmitted infections (STIs) including HIV, and to reduce the rate of under 18 conceptions, especially among vulnerable and at risk groups. NICE public health intervention guidance 3. 2007. London, NICE.
Ref Type: Report

23. Medical Foundation for AIDS & Sexual Health. Recommended standards for sexual health services. United Kingdom: 2005.

24. Rietmeijer C. Risk reduction counselling for prevention of sexually transmitted infections: how it works and how to make it work. Sexually Transmitted Infections 2007;83:2-9.

25. Waldo CR, Stall RD, Coastes TJ. Is offering post-exposure prevention for sexual exposures to HIV related to sexual risk behaviour in gay men? AIDS 2000;14:1035-9.

26. Rubak S, Sandboek A, Lauritzen T, Christensen B. Motivational interviewing: a systematic review and meta-analysis. British Journal of General Practice 2005;55:305-12.

27. Cosio D, Heckman TG, Anderson T, Heckman BD, Garske J, McCarthy J. Telephone-administered motivational interviewing to reduce risky sexual behavior in HIV-infected rural persons: A pilot randomized clinical trial. Sexually Transmitted Diseases 2010;37:140-6.

28. Fisher JD, Fisher WA, Cornman DH, Amico RK, Bryan A, Friedland GH. Clinician-delivered intervention during routine clinical care reduces unprotected sexual behaviour among HIV-infected patients. Journal of Acquired Immune Deficiency Syndromes 2006;41:44-52.

29. Fisher JD, Fisher WA. Changing AIDS risk behaviour. Psychological Bulletin 1992;111:455-74.

30. Miller, W. R. The Motivational Interviewing Skill Code (MISC) Manual, version 1. 2000. Available at: http://casaa.unm.edu/download/misc1.pdf. 15-4-2010.
Ref Type: Report

31. British Psychological Society Health Psychology Team. Improving Health: Changing Behaviour, NHS Health Trainer Handbook. London: Department of Health; 2008.

32. Morisky D, Ang A, Krousel-Wood M, Ward H. Predictive validity of a medication adherence measure in an outpatient setting. Journal of Clinical Hypertension 2008;10(5):348-54.

33. Misovich S, Fisher W, Fisher J. A measure of AIDS prevention information, motivation, behavioral skills, and behavior. In: Davis C, Yarber W, Bauserman R, et al, editors. Handbook of Sexuality-Related Measures. Thousand Oaks, CA: SAGE; 1998. p. 328-37.

34. Sayer C, Fisher M, Nixon E, Nambiah K, Richardson D, Perry N, Llewellyn C. Will I, won't I? Why do MSM present for PEPSE? Sexually Transmitted Infections 2009;**85**:206-11.
